# Single-cell RNA sequencing analysis reveals a lack of CXCL13^+^ T cell subsets associated with the recurrence of cervical squamous cell carcinoma following concurrent chemoradiotherapy

**DOI:** 10.1007/s00262-025-04083-3

**Published:** 2025-06-04

**Authors:** Xia Li, Yanmei Cheng, Mei Ji, Junqi Liu, Zhao Zhao, Qitai Zhao

**Affiliations:** 1 Department of Radiation Oncology, The First Affiliated Hospital of Zhengzhou University, Zhengzhou, 450052 Henan Province People’s Republic of China; 2 Department of Gynecology and Obstetrics, The First Affiliated Hospital of Zhengzhou University, Zhengzhou, 450052 Henan Province People’s Republic of China; 3 Biotherapy Center and Cancer Center, The First Affiliated Hospital of Zhengzhou University, Zhengzhou, 450052 Henan Province People’s Republic of China

**Keywords:** Cervical squamous cell carcinoma, Concurrent chemoradiotherapy, Recurrence, CXCL13^+^ T cells, Cell communication, Random forest model

## Abstract

Concurrent chemoradiotherapy (CCRT) is the standard treatment for advanced cervical cancer, but tumor recurrence within 3–5 years remains a significant challenge. In this study, using 10 × single-cell sequencing, we constructed a cellular atlas of the tumor microenvironment from six CSCC patients, including three with recurrence and three without, prior to CCRT. We analyzed cellular subsets, focusing on T cells, myeloid cells and cancer associated fibroblasts (CAFs), and their interactions within the tumor. Key findings revealed that CXCL13^+^ T cell subsets were significantly increased in non-recurrent tumors and acted as major signal senders. In recurrent tumors, FOXP3^+^ and IL2RA^+^ Tregs were the primary mediators of cell communications. CXCL13^+^ CD8^+^ T cells interacted with SPP1^+^ tumor-associated macrophages (TAMs) in non-recurrent tumors, while in recurrent tumors, they interacted with CD163^+^ TAMs. Moreover, in recurrent tumor tissues, this subset demonstrates a preferential interaction with MMP3^+^ CAFs. The study also identified five genes (PDCD1, CXCL13, TOX, RGS1, and ALOX5AP) based on CXCL13^+^ T cell signature to construct predictive models for recurrence, with the random forest model showing the best performance. This study provides new insights into recurrence mechanisms in CSCC and suggests that increasing CXCL13^+^ T cells could be a potential therapeutic strategy.

**Supplementary Information:**

The online version contains supplementary material available at 10.1007/s00262-025-04083-3.

## Introduction

Cervical cancer is the fourth most common cancer among women worldwide and a leading cause of cancer-related mortality, particularly in low- and middle-income countries. Cervical squamous cell carcinoma (CSCC) is the most prevalent subtype [1]. For locally advanced cervical cancer, concurrent chemoradiotherapy (CCRT) is the standard treatment. While CCRT is effective at achieving initial tumor control, 30–40% of patients experience recurrence within 3–5 years, which is associated with poor prognosis and limited therapeutic options [2, 3]. The mechanisms underlying recurrence are complex, involving both tumor-intrinsic factors and tumor microenvironment (TME)-associated processes, such as immune suppression and therapeutic resistance [4].

Single-cell RNA sequencing (scRNA-seq) has emerged as a powerful tool to investigate the TME with high resolution, enabling the identification of cellular heterogeneity and intercellular interactions. This technology provides critical insights into the molecular mechanisms driving treatment responses and resistance [5]. Recent scRNA-seq studies in cervical cancer have revealed the cellular diversity within tumor and adjacent non-tumor tissues, as well as during tumor progression [6–8]. Beyond its direct cytotoxic effects on tumor cells, CCRT significantly modulates the TME by inducing immunogenic cell death, enhancing tumor antigen release, and promoting immune cell infiltration [9]. Recent studies have explored the immune microenvironment changes in cervical cancer following CCRT, identifying key cellular populations such as MHC class II-expressing epithelial cells and atypical chemokine receptor 2^+^ tumor cells, which are associated with treatment response [10, 11]. However, the pre-existing cellular landscape of tumor tissues and its relationship with recurrence in cervical cancer patients undergoing CCRT remains poorly understood.

In this study, we performed single-cell RNA sequencing on pre-treatment tumor tissues from six CSCC patients, including three who experienced recurrence and three who did not. We identified a higher proportion of CXCL13⁺ T cells in non-recurrent tumors and investigated their interactions with myeloid cells and cancer associated fibroblasts to uncover potential mechanisms underlying recurrence. Additionally, we developed a predictive model using a random forest algorithm based on CXCL13⁺ T cell features. Our findings provide novel insights into the cellular landscape and recurrence mechanisms of CSCC following CCRT at the single-cell level.

## Materials and methods

### Patients and sample collection

Six patients diagnosed with HPV-positive cervical squamous cell carcinoma (CSCC) were included in this study. Tissue samples were obtained via biopsy or surgery 1 week prior to treatment. All patients underwent concurrent chemoradiotherapy (CCRT), which consisted of pelvic external beam radiotherapy (30–40 Gy; 15–17 fractions) and chemotherapy with paclitaxel (135–175 mg/m^2^) followed by cisplatin (50–70 mg/m^2^). All six patients received treatment between April and July 2021. After treatment, all patients exhibited a partial response (60–90%) according RECIST 1.1 criteria. During the first 2 years post-treatment, all patients underwent gynecological examinations (including vaginal vault and cervical assessments) every 3 months, combined with pelvic MRI scans every 6 months and chest CT at approximately 12-month intervals. PET-CT was performed when clinical evaluation suggested potential recurrence. From year 3 onward, clinical follow-ups were conducted semiannually. All patients completed the 3-year follow-up, with three cases developing recurrences at distinct sites: pulmonary metastasis, pelvic recurrence, and vulvar recurrence.

### Isolation of single cells

Tumor specimens were immediately washed with pre-cooled PBS to remove residual blood, then cut into small pieces. Digestion was carried out with 10 mL of digestion medium containing 0.2% collagenase I/II, DNase I (Sigma), and 25 units of dispase in DMEM. The reaction was terminated using 5% fetal bovine serum, and the mixture was centrifuged at 1500 rpm for 5 min. Red blood cells were lysed using 5 mL of red blood cell lysis buffer at room temperature for 10 min. The remaining cells were centrifuged, re-suspended in sorting buffer, and assessed for viability prior to use.

### Single-cell sequencing, filtering, and normalization

Single-cell libraries were prepared using the Chromium Single Cell 3' Library, Gel Bead & Multiplex Kit, and Chip Kit (10 × Genomics) according to the manufacturer's instructions. Raw data were processed into unique molecular identifier (UMI) counts using CellRanger (v3.0.2, 10 × Genomics) with the GRCh38 reference genome. Public scRNA data set of CSCC received CCRT treatment were downloaded from Gene Expression Omnibus (https://www.ncbi.nlm.nih.gov/geo/) with accession number (GSE236738) [11]. Downstream analysis was performed using the Seurat package (version 4.2.1) in R (version 4.0.1). Cells were filtered based on the following criteria: nFeature RNA > 7500, nCount RNA > 15,000, mitochondrial (MT) gene ratio > 25%, ribosomal protein (RP) gene ratio > 10%, and hemoglobin gene ratio (HB) > 40%. Following filtration, 37,949 high-quality cells were retained for further analysis. Data normalization was conducted using the “LogNormalize” method, and 2000 highly variable genes were identified using variance stabilizing transformation for principal component analysis (PCA).

### Unsupervised cell clustering and cell type annotation

To integrate single-cell data across samples, Harmony was applied, followed by clustering analysis at a resolution of 0.6. Dimensionality reduction was performed using Uniform Manifold Approximation and Projection (UMAP). Major cell populations were annotated based on canonical marker genes, leading to the identification of 12 major cell types. T cells and myeloid cells were isolated for further analysis using the subset function. For T cells, clustering was refined at a resolution of 1.5, while a resolution of 1 was applied for myeloid cells. Marker genes for each subpopulation were identified using the FindAllMarkers function with thresholds of *p*-value < 0.05 and log fold change > 0.5. The top five marker genes for each subpopulation were visualized using the R package ComplexHeatmap (version 2.12.0).

### Cell communication analysis within T cells and myeloid cells

The R packages CellChat (version 1.4.0) was used to analyze interactions within T cells and between T cells and myeloid cells. Cell subsets with fewer than 200 cells were excluded, resulting in 10 subsets divided into recurrent and non-recurrent groups. “Secreted Signaling” and “Cell–Cell Contact” pathways were analyzed. Ligand-receptor pairs with fold change > 0.25 and *p*-value < 0.05 were identified and mapped to a protein–protein interaction (PPI) network. Interaction networks between subsets were visualized using the netVisual_circle function. To compare T cell interactions between recurrent and non-recurrent tumors, interaction data from both groups were integrated, and the compareInteractions function was used to assess overall interaction numbers and strengths. The netVisual_diffInteraction function was applied to visualize differences, where blue lines represent increased interactions in recurrent tumors and red lines indicate increased interactions in non-recurrent tumors. Differences in signaling pathways were analyzed using the ranknet function, and the top five signaling pathways were visualized with a heatmap. The netAnalysis_signalingRole_scatter function identified the main signal senders and receivers, while the netAnalysis_signalingChanges_scatter function was used to examine specific signaling pathways of CXCL13^+^ CD4 and CD8 subsets in both tumor types. For myeloid cell analysis, clusters with fewer than 100 cells were excluded, resulting in nine subsets, and interaction parameters were set consistent with T cell analysis.

### Cell communication analysis between CXCL13^+^ T cells and myeloid cells

To investigate interactions between CXCL13^+^ T cells and myeloid cells, the nine myeloid subsets were integrated with CXCL13^+^ T cell subsets. Interaction analysis parameters were consistent with previous analyses. Specific outgoing and incoming signals for CXCL13^+^ CD4 and CD8 cells in recurrent and non-recurrent tumors were compared using the netAnalysis_signalingChanges_scatter function. The netVisual_aggregate function visualized these specific signals for CXCL13^+^ CD4 and CD8 subsets.

### Construction and validation of the predictive model

To construct the predictive model, we selected maker genes to represent cell type [12, 13]. we first identified the intersection of maker genes with a log fold change > 1 in the c10-CXCL13-CD8 and c3-CXCL13-CD4 subsets, resulting in 15 genes. RNA sequencing data for cervical cancer were downloaded from the TCGA database (UCSC Xena, http://xena.ucsc.edu/), and Spearman correlation analysis with CD8A was performed, selecting 5 genes with a correlation coefficient > 0.5 for model construction. R packages [caret (version 7.0.1), xgboost (version 1.7.8.1) and random forest (version 3.2.2)] were used to construct logistic regression (LR), random forest (RF), support vector machine (SVM), k-nearest neighbors (KNN), and gradient boosting machines (GBM) models. Model performance was evaluated by calculating the area under the curve (AUC) using the R package pROC (version 1.18.0). For validation, immunotherapy datasets were obtained from the Tumor Immunotherapy Gene Expression Resource (http://tiger.canceromics.org/) [14], and a random forest model was constructed using the 5 selected genes. Additionally, cervical cancer mRNA sequencing data and survival data were downloaded from UCSC Xena, and a random forest model was applied for further validation.

### Estimation of signaling pathways

To analysis the signaling difference in CAFs between recurrent and non-recurrent tumors, we first downloaded the hallmark gene set from the Gene Set Enrichment Analysis website (https://www.gsea-msigdb.org/). R package GSVA (version 1.44.0) was used to estimate the score of signaling in each samples. R package limma (version 3.52.1) was used to calculate difference between recurrent and non-recurrent group. Significant signaling was defined as *p* < 0.05 and* t* > 5.

### Multicolor immunofluorescence

The tissue sections were baked at 65 °C for 2 h in a slide dryer, followed by deparaffinization and rehydration through xylene and graded alcohol series. Antigen retrieval was then performed using pH 8.0 EDTA alkaline buffer, with subsequent endogenous peroxidase blocking by 3% hydrogen peroxide treatment. After removing the blocking buffer, primary antibodies against pan-cytokeratin (pan-CK, AF20164, AiFang biological), CD3(AF20162, AiFang biological), and PD-1(AF20083, AiFang biological) were applied simultaneously and incubated overnight at 4 °C. Following primary antibody incubation, sections were washed three times with PBST (Phosphate-Buffered Saline with 0.1% Tween-20) and subsequently incubated with species-matched secondary antibodies for 30 min at room temperature. After another three PBST washes, tyramide signal amplification (TSA) dyes were applied for 3–10 min (wavelength-specific incubation times optimized per fluorophore). Post-staining, nuclei were stained with DAPI (4′,6-diamidino-2-phenylindole; 1 μg/mL, 5 min), and slides were mounted with antifade medium prior to imaging using a laser scanning confocal microscope.

### Reverse transcription (RT)-PCR analysis

Frozen CESC tumor tissues stored in the biobank were cut into small pieces and placed into 2 mL EP tubes. RNA was extracted using RNAiso Plus (TaKaRa, #D9108A) according to the manufacturer’s protocol. Briefly, 1 mL of RNAiso Plus was added to the tissue, followed by 500 µL of chloroform. The lysate was mixed thoroughly and centrifuged at 12,000 rpm for 15 min at 4 °C. The colorless upper phase was collected, mixed with an equal volume of isopropanol, and incubated on ice for 10 min. The mixture was then centrifuged at 12,000 rpm for 10 min at 4 °C. The RNA pellet was washed three times with pre-cooled 75% ethanol, centrifuged at 12,000 rpm for 5 min at 4 °C, air-dried, and dissolved in RNase-free water. RNA concentration was measured using a NanoDrop 2000. A total of 1 µg RNA was reverse-transcribed into cDNA using the HiScript II Q RT SuperMix (Vazyme, #R333-01) according to the manufacturer’s instructions. Gene-specific primers used in this study are listed in Table S1. Relative expression levels of target genes were calculated using the 2^(-ΔΔCt) method, with *GAPDH* serving as the internal control. Each sample was analyzed in triplicate to ensure accuracy and reproducibility.

### Statistical analysis

All statistical analyses were performed using R software (version 4.2.1). The Wilcoxon test was used for comparisons between two groups, while ANOVA was applied for comparisons among three or more groups. Survival analyses were conducted using the log-rank test. A *p*-value < 0.05 was considered statistically significant.

## Results

### Single-cell atlas identifies major subsets associated with recurrence after CCRT in patients with CSCC

**Fig. 1:**
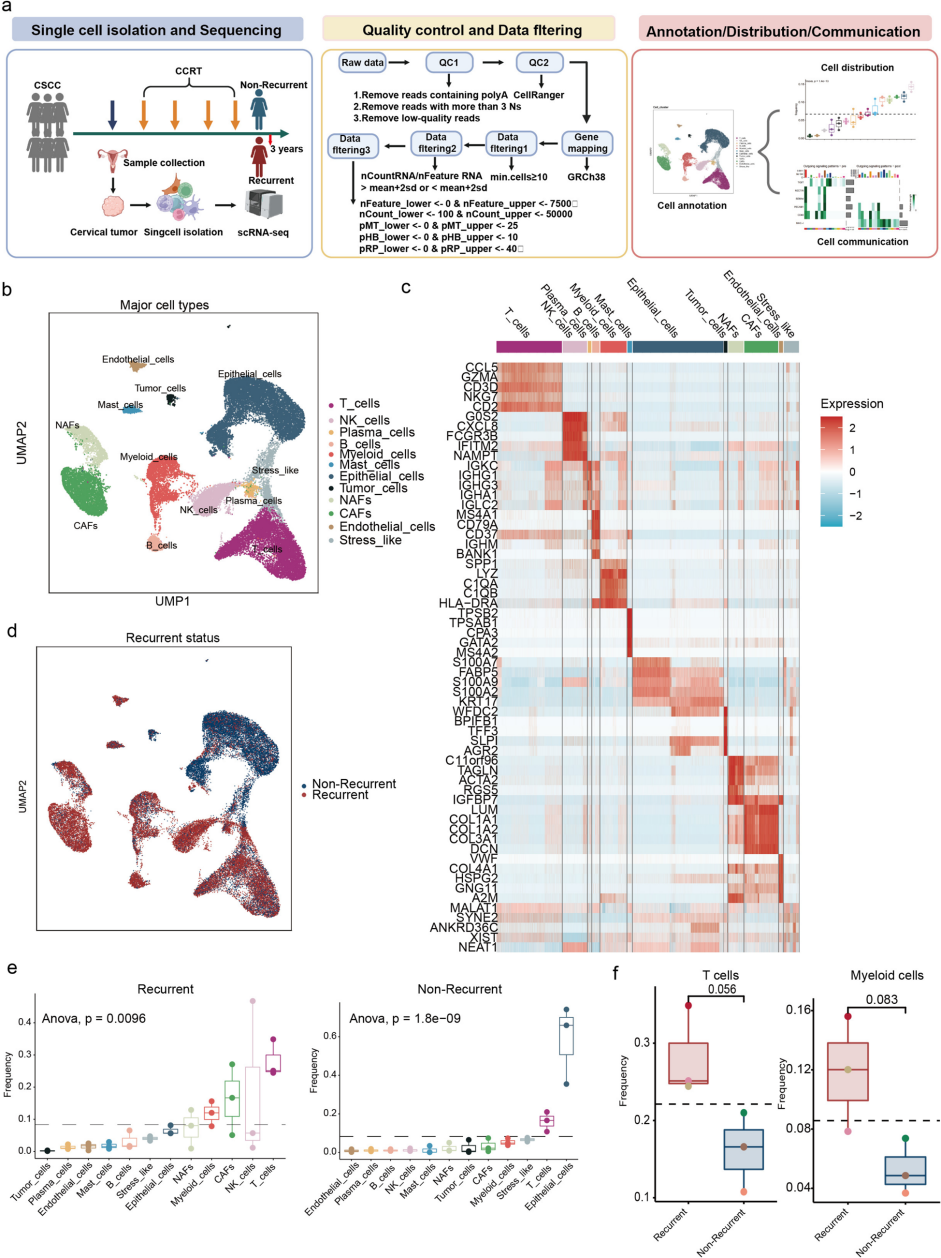
Landscape of cell landscape in CSCC tumor prior to CCRT.** a** Schematic diagram explaining the workflow of the experimental design. **b** UMAP displaying 43,566 cells from six CSCC tumor samples revealing 12 cell subsets. **c** Heatmap showing the top five maker genes of each subset. **d** UMAP plot showing the 12 cell subsets separated by recurrent status. **e** Boxplots showing the distribution of 12 cell subsets in recurrent and non-recurrent tumors. **f** Boxplots showing the ratios of T cells and myeloid cells in recurrent and non-recurrent tumors

To explore the factors contributing to tumor recurrence following CCRT, we collected tumor samples from six patients with CSCC prior to CCRT and performed single-cell RNA sequencing. Among these patients, three experienced tumor recurrence within 3 years, while the remaining three showed no signs of recurrence (Fig. 1a). Detailed clinical characteristics of the patients are provided in Table 1. Following stringent data filtering and integration across all samples, 43,566 high-quality cells were retained for downstream analyses (Fig. S1a–e). Using the uniform manifold approximation and projection (UMAP) algorithm, we clustered these cells into distinct groups. Based on canonical gene expression patterns, we classified the clusters into 12 major subsets, each characterized by distinct and specific gene expression profiles (Fig. 1b, c; Fig. S2a, b). Moreover, by analyzing public databases, we identified similar subpopulations in cervical cancer tissues (Fig. S2c–e). Notably, all subsets were detected in each tumor sample; however, their distribution varied significantly between recurrent and non-recurrent tumors (Figs. 1d, S3a). Further analysis revealed that T cells and myeloid cells were predominantly enriched in recurrent tumors, whereas epithelial cells were more abundant in non-recurrent tumors (Fig. 1e). Comparative analysis of cell proportions between the two groups demonstrated significant differences, particularly in the proportions of T cells and myeloid cells (Figs. 1f, S3b). These findings highlight the potential roles of immune and epithelial cell dynamics in tumor recurrence after CCRT treatment.

**Table 1:** Clinical information of included patients

Patients	Age	Stage	Differentiation	Tumor characteristics	Recurrent status in third year	Pathological type
PT1	68	IIA	Low	1. Nerve invasion	No recurrence	CSCC
				2. Multiple tumor thrombi in vascular spaces		
PT2	39	IIIA	Moderate	1. Retroperitoneal lymph node metastasis	Lung metastasis	CSCC
				2. Left iliac vascular lymph node metastasi		
PT3	57	IIB	Low	1. Intravascular tumor thrombus	Pelvic recurrence	CSCC
				2. Tumor size > 5 cm		
PT4	47	IIB	High	1. Cervical stromal invasion > 1/3	No recurrence	CSCC
				2. Multiple tumor thrombi in vascular spaces		
				3. Tumor size > 3 cm		
PT5	39	IB	Low	1. Nerve invasion	No recurrence	CSCC
				2. Cervical stromal invasion > 1/3		
				3. Tumor size > 3 cm		
PT6	42	IB	Moderate	1. Tumor size > 3 cm	Vulvar recurrence	CSCC
				2. Multiple tumor thrombi in vascular spaces		

### CXCL13^+^ T cells are enriched in non-recurrent tumors following CCRT treatment in patients with CSCC

**Fig. 2:**
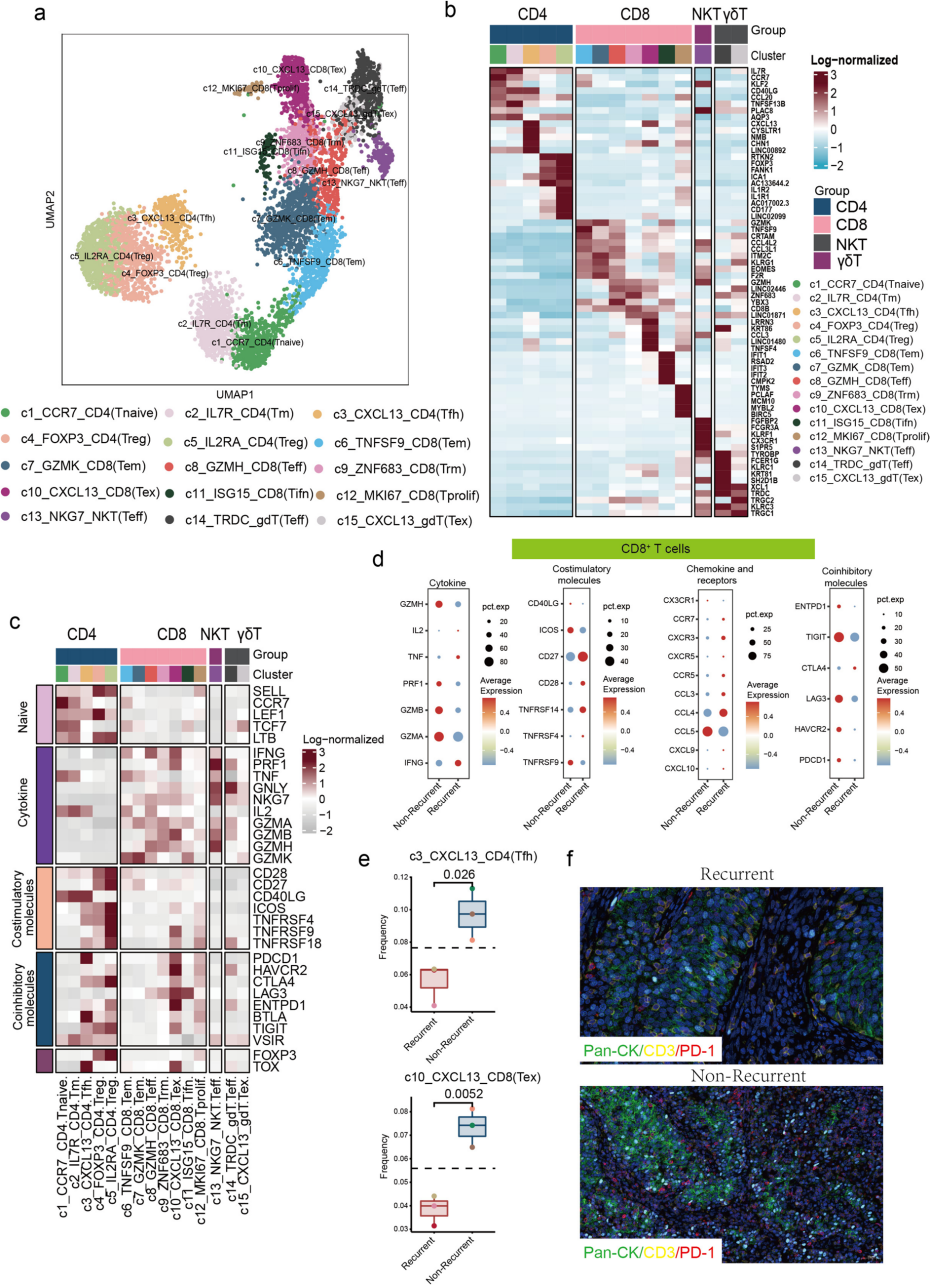
Characterization of T cells in CSCC tumor prior to CCRT. **a** UMAP displaying 15 cluster of T cell subsets. **b** Heatmap showing the top five maker genes of each T cell subset. **c** Heatmap showing functional gene expression, including naive, cytotoxic, co-stimulatory and co-inhibitory genes, across T cells subsets. **d** Dot plot showing functional gene expression, including cytotoxic, co-stimulatory, chemokine and receptor and co-inhibitory genes, in CD8^+^ T cells. **e** Boxplots showing the ratios of c3-CXCL13-CD4 (Tfh) and c10-CXCL13-CD8(Tex) in recurrent and non-recurrent tumors. **f** Multicolor IF staining in two representative CESC tumor from recurrent and non-recurrent patients prior to CCRT

Further clustering analysis of T cells identified five CD4^+^ T cell subsets and seven CD8^+^ T cell subsets, characterized primarily by the expression of the *CD3D*, *CD4*, and *CD8A* genes, respectively (Figs. 2a, S4a). Additionally, one NKT cell sub-cluster, defined by the expression of *FCGR3A*, and two γδ T cell subsets, marked by the expression of *TRDC*, were identified. These cell subsets were distributed across the samples (Figs. 2a, S4b), with each subset exhibiting distinct gene expression signatures (Fig. 2b). Further functional analysis revealed that the CD4^+^ T cell subsets displayed higher expression of genes associated with naïve T cells and co-stimulatory molecules, while CD8^+^ T cell subsets demonstrated elevated expression of cytokine-related genes, indicating their distinct functional roles within the tumor microenvironment (Fig. 2c). In non-recurrent tumors, CD8^+^ T cells exhibited high expression levels of functional molecules, including *GZMH*, *PRF1*, *GZMB*, and *GZMA*, as well as co-stimulatory molecules, such as *CD40LG*, *ICOS*, and *TNFRSF9*. Additionally, these cells also expressed inhibitory molecules, including *ENTPD1*, *TIGIT*, *LAG3*, *HAVCR2*, and *PDCD1* (Fig. 2d). Consistent with the findings in CD8^+^ T cells, CD4^+^ T cells in non-recurrent tumors also exhibited significantly higher expression of functional and co-stimulatory molecules (Fig. S4c). Notably, non-recurrent tumors had a high level of immunosuppressive CD4^+^ T cell subsets, including two Treg subsets. (Figs. S4d). By comparing the proportional differences between recurrent and non-recurrent tumor cells, we found that CXCL13^+^ CD4^+^ and CD8^+^ T cell subsets were enriched in non-recurrent tumors (Figs. 2e, S4e). These two cell populations were characterized by the expression of co-inhibitory molecules such as *PDCD1* and *HAVCR2* (Fig. 2b). Using multiplex immunofluorescence, we further demonstrated that the proportion of these cells was significantly increased in non-recurrent tumor tissues (Fig. 2f). Furthermore, we analyzed scRNA sequencing data from cervical cancer tumors before and after CCRT. We similarly identified these two cell populations and observed that the CXCL13^+^CD8^+^T subset tended to decrease in proportion post-treatment, while the CXCL13^+^CD4^+^T subset showed an increasing trend, although neither change reached statistical significance (Fig. S5a–c). These findings suggest that the enrichment of CXCL13^+^ T cells and their functional activity may play a critical role in preventing tumor recurrence.

### Intra-interactions within T cells reveal associations with recurrence following CCRT treatment in patients with CSCC

**Fig. 3:**
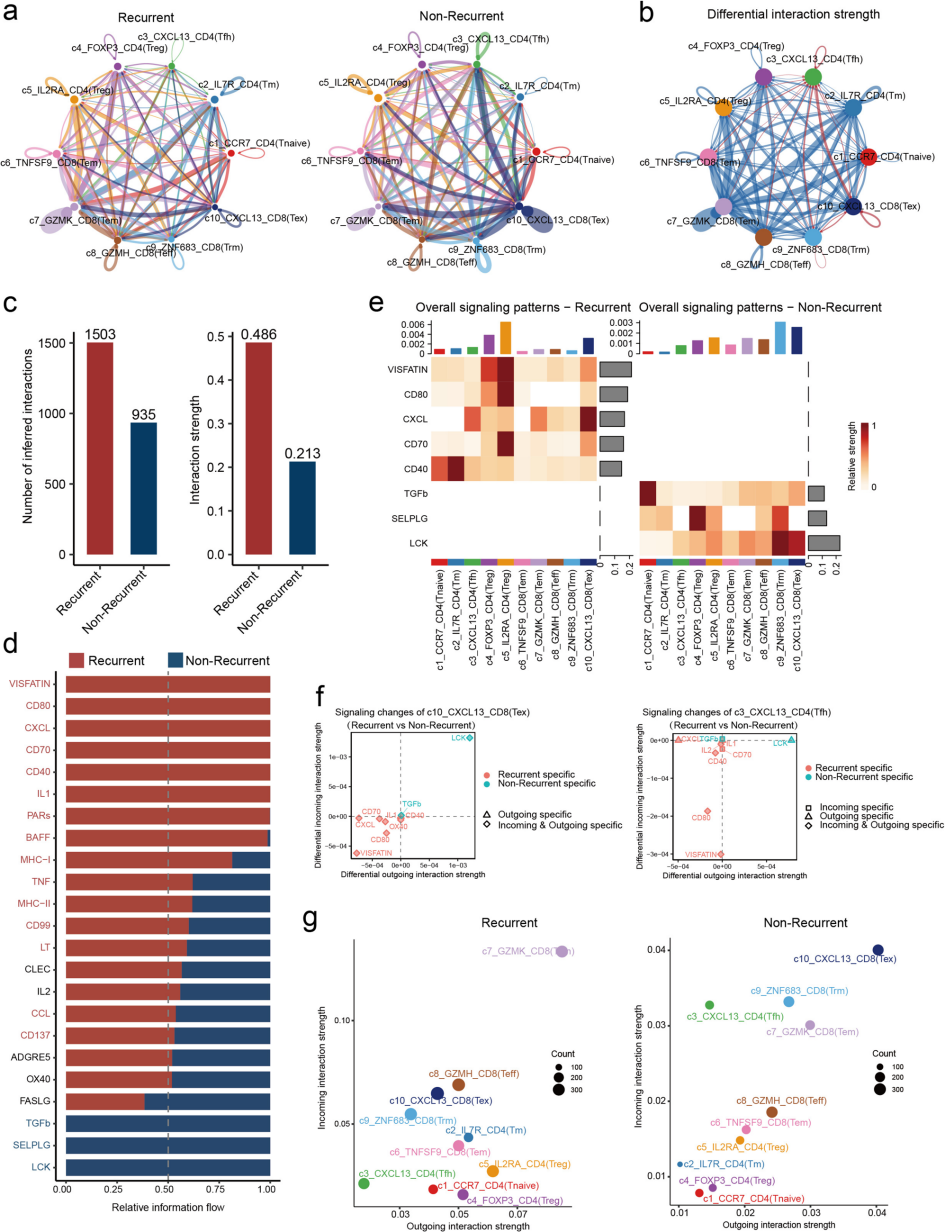
Cell communications within T cell subsets. **a** Circle plot showing interactions across T cell subsets in recurrent and non-recurrent tumors, respectively. **b** Circle plot showing the difference of interactions across T cell subsets between recurrent and non-recurrent tumors. Blue lines indicate enhanced interactions in recurrent tumors, while red lines indicate enhanced interactions in non-recurrent tumors. **c** Bar plot showing the number and strength of interactions across T cell subsets in recurrent and non-recurrent tumors. **d** Stacked plot showing different signaling in recurrent and non-recurrent tumors medicated interactions across T cell subsets. **e** Heatmap showing the top five signaling across T cell subsets in recurrent and non-recurrent tumors, respectively. **f** Scatter plot showing outgoing and incoming signaling specific to c10-CXCL13-CD8(Tex) and c3-CXCL13-CD4(Tfh), respectively. **g** Scatter plot showing the receiver and sender of T cells subsets in recurrent and non-recurrent tumors, respectively

Cell–cell communication plays a critical role in regulating cellular functions within the tumor microenvironment. To explore its role in recurrence, we analyzed T cell interactions in recurrent and non-recurrent tumors. In non-recurrent tumors, c10-CXCL13^+^ CD8^+^ T cells (Tex) exhibited the strongest interactions with c3-CXCL13^+^ CD4^+^ T cells (Tfh) and c7-GZMK^+^ CD8^+^ T cells (Tem). Conversely, in recurrent tumors, c4-FOXP3^+^ CD4^+^ T cells (Treg) and c2-IL7R^+^ CD4^+^ T cells (Tm) displayed the highest interaction intensities with other cells (Fig. 3a). Differential analysis of T cell interactions between recurrent and non-recurrent tumors revealed that c3-CXCL13^+^ CD4^+^ T cells (Tfh) and c10-CXCL13^+^ CD8^+^ T cells (Tex) dominated the communication network in non-recurrent tumors (Fig. 3b). Interestingly, the intensity and number of T cell interactions were significantly higher in recurrent tumors compared to non-recurrent tumors (Fig. 3c). Consistent with this, the signaling pathways mediating cell–cell communication exhibited higher intensities in recurrent tumors (Fig. 3d). These findings suggest a potentially more complex intercellular interaction network in recurrent tumors. Notably, the VISFATIN, CD80, CXCL, CD70, and CD40 pathways were significantly enhanced in recurrent tumors, whereas the LCK, SELPLG, and TGF-β pathways demonstrated stronger interaction intensities in non-recurrent tumors (Fig. 3e). Differential analysis of cell–cell communication involving c10-CXCL13^+^ CD8^+^ T cells (Tex) and c3-CXCL13^+^ CD4^+^ T cells (Tfh) between recurrent and non-recurrent tumors revealed that the LCK and TGF-β signaling pathways were specific to non-recurrent tumors. In c10-CXCL13^+^ CD8^+^ T cells (Tex), these pathways predominantly mediated both outgoing and incoming signals. For c3-CXCL13^+^ CD4^+^ T cells (Tfh), LCK primarily facilitated outgoing signals, while TGF-β was mainly involved in incoming signal interactions (Fig. 3f). Further analysis of the signal senders and receivers in recurrent and non-recurrent tumors revealed distinct patterns. In non-recurrent tumors, the primary senders and receivers were the c10-CXCL13^+^ CD8 subset, the c3-CXCL13^+^ CD4 subset, the c9-ZNF683^+^ CD8 (Trm) subset, and the c7-GZMK^+^ CD8 (Tem) subset. In contrast, in recurrent tumors, the c7-GZMK^+^ CD8 subset was the predominant sender and receiver. Notably, two inhibitory CD4 subsets, c4-FOXP3^+^ CD4 (Treg) and c5-IL2RA^+^ CD4 (Treg), emerged as the main signal senders in recurrent tumors (Fig. 3g). These findings underscore the distinct signaling dynamics and intercellular communication patterns within T cell subsets in recurrent versus non-recurrent tumors, highlighting their potential roles in tumor recurrence.

### Single-cell analysis of myeloid cell heterogeneity and its correlation with recurrence following CCRT treatment in patients with CSCC

**Fig. 4:**
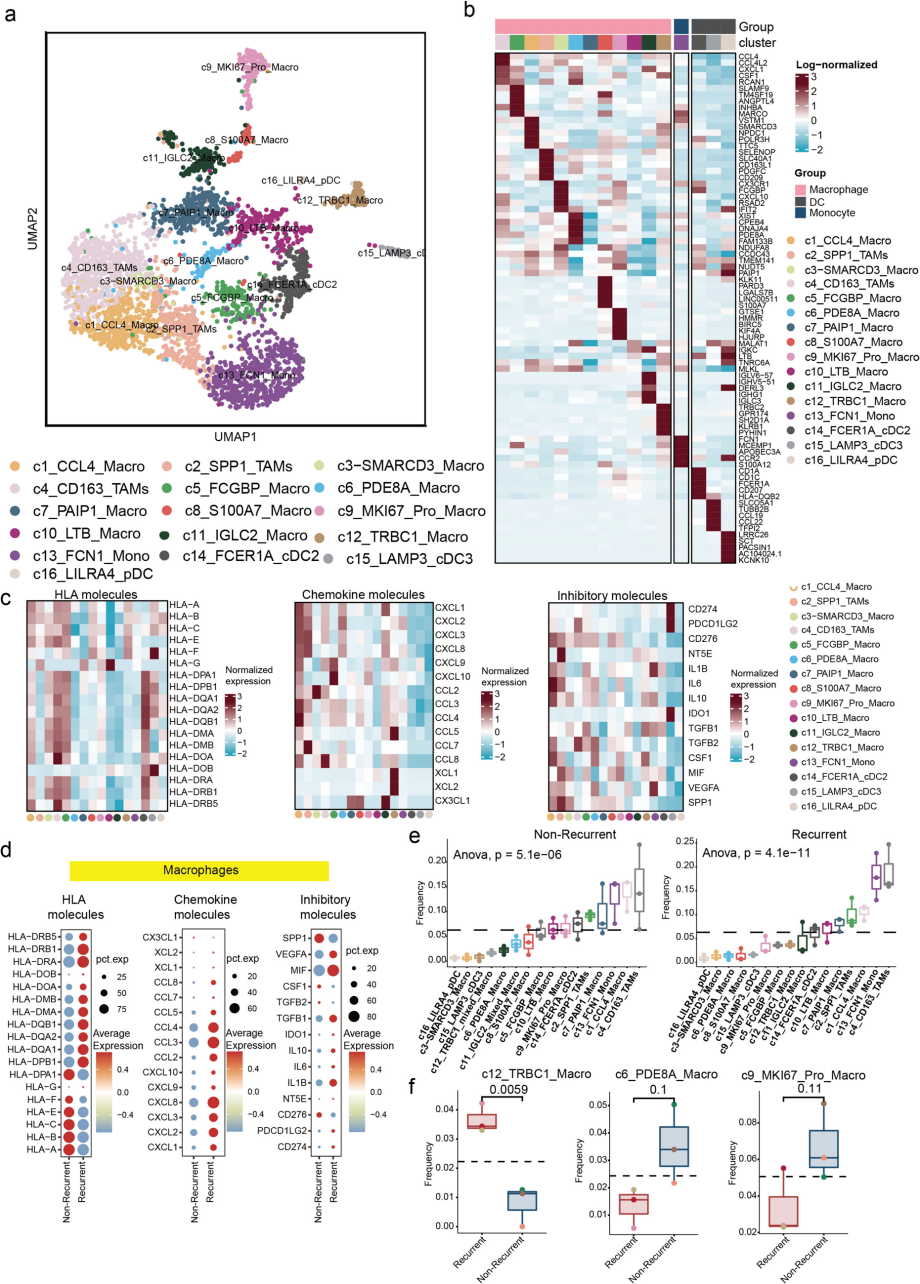
Characterization of myeloid cells in CSCC tumor prior to CCRT. **a** UMAP displaying 16 cluster of myeloid cell subsets. **b** Heatmap showing the top five maker genes of each myeloid cell subset. **c** Heatmap showing the gene expression related to HLA molecules, chemokines and immunosuppressive genes in myeloid subsets. **d** Dot plot showing expression of HLA molecules, chemokines and immunosuppressive genes in macrophages subsets. **e** Boxplots showing the distribution of 16 myeloid cell subsets in recurrent and non-recurrent tumors, respectively. **f** Boxplots showing the ratios of c12-TRBC1-Macro, c6-PDE8A-Macro and c9-MKI67-Pro-Macro in recurrent and non-recurrent tumors

To investigate the heterogeneity of myeloid cells, we performed clustering analysis and identified a total of 16 subsets. Based on classical gene expression profiles, these subsets were classified into three major groups (Fig. S6a): macrophages, which formed the dominant group with 12 subsets; one monocyte subcluster; and three dendritic cell (DC) subsets (Fig. 4a). Each subcluster displayed distinct gene expression signatures (Fig. 4b). Although all subsets were present in each sample, their proportions varied significantly between recurrent and non-recurrent tumors (Figs. S6b, c). Among these subsets, we identified that c4-CD163-Tumor associated macrophages (TAMs) and c14-FCER1A-cDC2 exhibited high expression of human major histocompatibility complex class I (MHC-I) molecules. The c1-CCL4-Macro subcluster showed elevated expression of chemokines, while the c1-CCL4-Macro, c2-SPP1-Macro, and c15-LAMP3-cDC3 subsets expressed high levels of immunosuppressive molecules, including PD-L1, IDO1, and SPP1 (Fig. 4c). Further analysis revealed distinct functional differences in macrophage subsets between recurrent and non-recurrent tumors. In non-recurrent tumors, macrophage subsets exhibited higher expression of MHC-I molecules but reduced expression of MHC-II molecules and chemokines. In contrast, macrophage subsets from recurrent tumors displayed significantly elevated expression of immunosuppressive molecules (Fig. 4d). Analysis of the distribution of myeloid cell subsets in recurrent and non-recurrent tumors revealed that the c4-CD163-Macro subcluster was the most abundant in both groups, with a similar distribution pattern observed among the top subsets in each group (Fig. 4e). However, further comparison of subsets differences between recurrent and non-recurrent tumors revealed that c12-TRBC1-Macro was enriched in recurrent tumors, whereas c6-PDE8A-Macro and c9-MKI67-Pro-Macro were predominantly found in non-recurrent tumors (Figs. 4f, S6d). These findings highlight the functional diversity of myeloid cell subsets and their potential roles in modulating the tumor microenvironment, particularly in relation to tumor recurrence following CCRT treatment.

### Interaction between CXCL13^+^ T cells and myeloid cells correlates with tumor recurrence following CCRT treatment in patients with CSCC

**Fig. 5:**
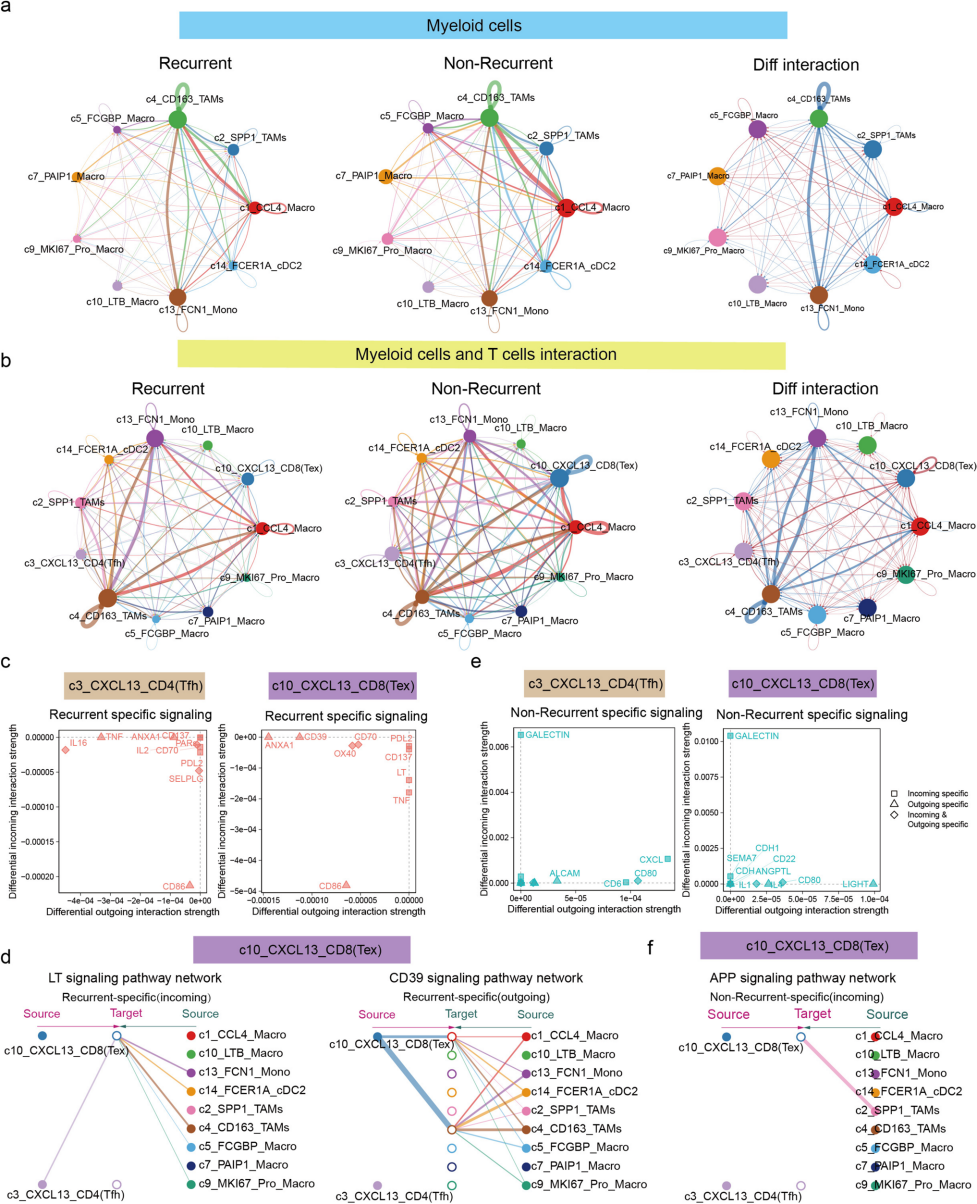
Interactions between CXCL13^+^ T cell subsets and myeloid subsets in CSCC tumor prior to CCRT. **a** Circle plot showing interactions across myeloid cell subsets in recurrent, non-recurrent tumors, as well as different interactions between recurrent and non-recurrent tumors, respectively. **b** Circle plot showing interactions of c10-CXCL13-CD8(Tex), c3-CXCL13-CD4(Tfh) and myeloid cell subsets in recurrent and non-recurrent tumors, as well as different interactions between recurrent and non-recurrent tumors, respectively. **c** Scatter plot showing outgoing and incoming signaling specific to c3-CXCL13-CD4(Tfh) and c10-CXCL13-CD8(Tex) subsets in recurrent tumors. **d** Hierarchy plot showing receivers and senders of LT and CD39 signaling specific to c10-CXCL13-CD8(Tex) subset in recurrent tumors. **e** Scatter plot showing outgoing and incoming signaling specific to c3-CXCL13-CD4(Tfh) and c10-CXCL13-CD8(Tex) subsets in non-recurrent tumors. **f** Hierarchy plot showing receivers and senders of APP signaling specific to c10-CXCL13-CD8(Tex) subset in non-recurrent tumors

Myeloid cells are the primary antigen-presenting cells in the tumor microenvironment, promoting T cell activation. Therefore, we first analyzed the interactions among myeloid cell subsets. Our results revealed that interactions involving c4-CD163-TAMs, c1-CCL4-Macro, and c13-FCN1-Mono played a dominant role in both recurrent and non-recurrent tumors. Further analysis showed that in recurrent tumors, primary interactions occurred between c4-CD163-TAMs and c13-FCN1-Mono, while in non-recurrent tumors, c5-FCGBP-Macro and c14-FCER1A-cDC2 contributed more significantly to intercellular communication (Fig. 5a). Additionally, we found that myeloid cells in recurrent tumors exhibited stronger interaction intensities and a greater number of interactions compared to those in non-recurrent tumors (Fig. S7a). Signaling pathway analysis revealed that CD99, ITGB2, and TGF-β signaling were upregulated in recurrent tumors, whereas PECAM1, APP, and IL-1 signaling were enriched in non-recurrent tumors (Fig. S7b). Further analysis revealed that CXCL13^+^ CD4 and CD8^+^ T cell subsets exhibited more frequent interactions with myeloid cells in non-recurrent tumors. Notably, we observed a strong interaction between c1-CCL4-Macro and c10-CXCL13-CD8 (Tex) in non-recurrent tumors (Fig. 5b). In recurrent tumors, we identified nine specific signaling pathways shared by the CXCL13^+^ CD4 and CD8 subsets that mediated intercellular communication (Fig. S8a). Among these, the LT signaling pathway was a unique incoming pathway for the CXCL13^+^ CD8 subsets, primarily receiving signals from c13-FCN1-Mono and c4-CD163-TAMs. Additionally, CD39 was identified as the major outgoing signal from the c10-CXCL13^+^ CD8 subset, targeting primarily c1-CCL4-Macro and c4-CD163-TAMs (Fig. 5c, d). The OX40 signaling pathway was identified as the primary mediator of outgoing and incoming interactions between the c10-CXCL13-CD8 and c3-CXCL13-CD4 subsets (Fig. S8b). For the c3-CXCL13^+^ CD4 subset, IL16, SELPLG, and IL2 were identified as its primary signaling pathways (Fig. S8c, d). In non-recurrent tumors, we identified ten commonly activated signaling pathways shared by the CXCL13^+^ CD4 and CD8 subsets (Fig. S8a). Notably, the APP signaling pathway was a specific incoming pathway for the c10-CXCL13^+^ CD8 subset, with c2-SPP1-TAMs serving as the primary signal source. Furthermore, we identified five signaling pathways specifically mediating the communication of the c3-CXCL13-CD4 subset, namely the ALCAM, CD6, CXCL, OSM, and SN pathways (Fig. S8e). These findings highlight the differential interaction networks between CXCL13^+^ T cells and myeloid cells in recurrent versus non-recurrent tumors, providing valuable insights into the mechanisms underlying tumor recurrence following CCRT treatment in patients with CSCC.

### CXCL13^+^ T cell subsets interacts with MMP3^+^ CAFs in recurrent tumor

**Fig. 6:**
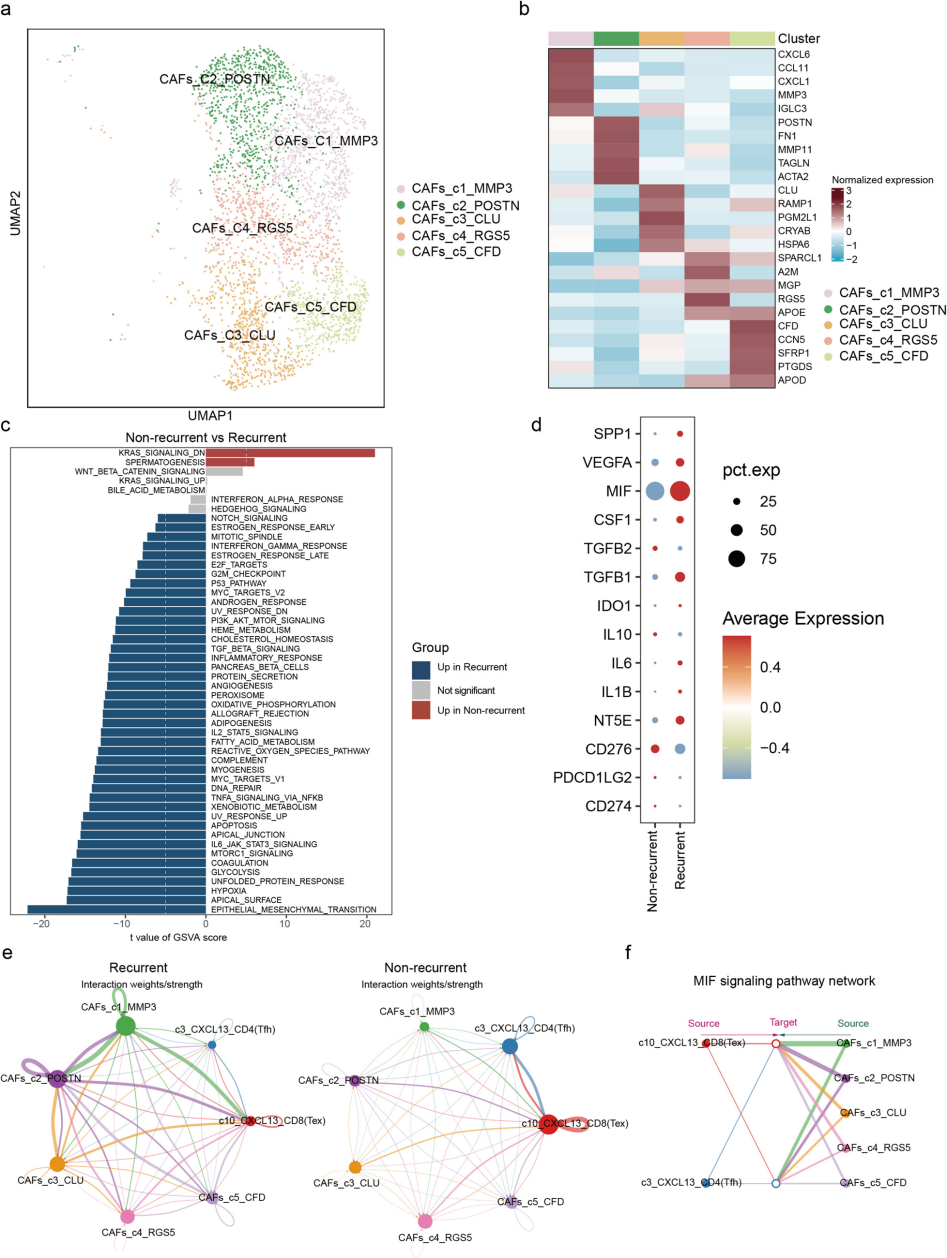
Characterization and interaction of CAFs subsets prior to CCRT. **a** UMAP displaying five subsets of CAFs. **b** Heatmap showing the top five maker genes of each CAFs cell subset. **c** Barplot showing up-regulated oncogenic signaling pathways in CAFs between recurrent and non-recurrent tumors. **d** Dot plot showing the immunosuppressive gene expression in CAFs between recurrent and non-recurrent tumors. **e** Circle plot showing interactions of c10-CXCL13-CD8(Tex), c3-CXCL13-CD4(Tfh) and CAFs subsets in recurrent and non-recurrent tumors, respectively. **f** Hierarchy plot showing receivers and senders of MIF signaling specific to c3-CXCL13-CD4(Tfh) and c10-CXCL13-CD8(Tex) subsets in CESC

CAFs play critical roles in tumorigenesis, tumor progression, and tumor immunity [15]. Our preliminary findings suggest an increased abundance of CAFs in recurrent tumors (Fig. S3b). To further investigate the role of CAFs in CSCC recurrence, we performed re-clustering of CAFs and identified five distinct subpopulations, each expressing unique marker genes (Fig. 6a, b). In recurrent tumor tissues, the proportions of two distinct CAFs subsets (CAFs-c2-POSTN and CAFs-c1-MMP3) were significantly elevated (Fig. S9a). However, none of the subsets showed statistically significant differences between recurrent and primary tumor tissues (Fig. S9b). Further analysis revealed that CAFs in recurrent tumors exhibited significant activation of multiple pro-tumorigenic pathways, including Hypoxia, glycolysis, and IL6/JAK/STAT3 signaling (Fig. 6c). Multiple immunosuppressive molecules were also highly expressed in CAFs from recurrent tumors (Fig. 6d). Analysis of intercellular interactions between CAF subsets and CXCL13^+^ T cell subsets revealed significantly stronger crosstalk between CAFs-c1-MMP3 subsets and CXCL13^+^ T cells, which was primarily mediated through the macrophage migration inhibitory factor (MIF) signaling pathway (Figs. 6e, f).

### CXCL13^+^ T cell signature predicts recurrence in CESC patients undergoing CCRT and response to immunotherapy in solid tumors

**Fig. 7:**
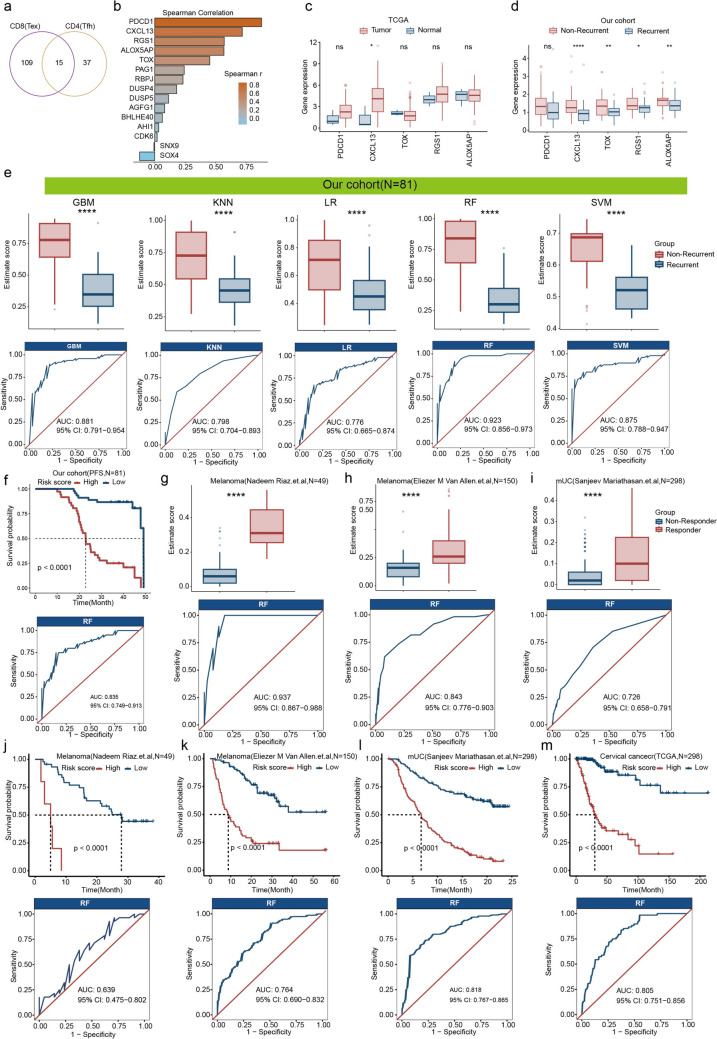
Construction and validation of predictive model. **a** Intersection of maker genes between c3-CXCL13-CD4(Tfh) and c10-CXCL13-CD8(Tex) subsets.** b** Bar plot showing the correlation of CD8A and intersected genes.** c** Boxplot plot showing the expression of signature genes between tumor and normal tissues of cervical cancer in TCGA database. **d** Boxplot plot showing the expression of signature genes between recurrent and non-recurrent tumors of cervical cancer in our cohort. **e** Boxplot (upper) and ROC (lower) analysis showing the estimated score based on signature genes by GBM, KNN, LR, RF and SVM between recurrent and non-recurrent tumor tissues. **f** K-M plot (upper) and ROC (lower) analysis showing PFS of high and low risk groups prior to CCRT and AUC of RF predictive model in our cohort. **g-i** Boxplot (upper) and ROC (lower)analysis showing the estimated score between responders and non-responders with immunotherapy treatment and AUC of RF predictive model in two melanoma datasets and mUC dataset. **j-l** K-M plot (upper) and ROC (lower)analysis showing survival of high and low risk groups with immunotherapy treatment and AUC of RF predictive model in two melanoma datasets and mUC dataset. **m** K-M plot (upper) and ROC (lower)analysis showing survival of high and low risk groups of cervical patients in TCGA database and AUC of RF predictive model

To evaluate whether CXCL13^+^ CD4^+^ and CD8^+^ T cell subsets can predict recurrence in CESC patients undergoing CCRT, we first identified the intersection of signature genes from these two cell subsets, yielding a total of 15 genes (Fig. 7a). Subsequently, correlation analysis between these genes and *CD8A* expression was performed, resulting in the selection of 5 genes with a correlation coefficient greater than 0.5 to form the predictive gene set (Fig. 7b). Analysis of these signature genes showed an overall upregulation trend in tumor tissue, with *CXCL13* exhibiting significantly higher expression in tumor compared to normal tissues (Fig. 7c). Moreover, the expression of these genes was significantly elevated in non-recurrent tumors (Fig. 7d). Using these five genes, we constructed predictive models utilizing five different machine learning methods. The results demonstrated that the gene set scores predicted by all models were significantly higher in non-recurrent tumors. Among these models, the random forest model exhibited the best performance, achieving an area under the curve (AUC) of 0.923(Fig. 7e). Moreover, this model demonstrated excellent predictive performance for progression-free survival, with an AUC value of 0.835(Fig. 7f). These findings indicate that the five-gene signature serves as a robust predictor of recurrence in CESC patients undergoing CCRT. Previous studies have reported an association between CXCL13^+^ CD8^+^ T cells and response to immunotherapy [16]. To investigate whether our model could predict immunotherapy efficacy, we validated it using two melanoma datasets and one metastatic urothelial carcinoma dataset, all from patients receiving immunotherapy. The results showed that the model effectively distinguished between responders and non-responders to immunotherapy, with AUC values of 0.937, 0.843, and 0.726, respectively (Fig. 7g–i). Additionally, the prognostic model based on the five-gene signature revealed that patients with higher risk scores had poorer prognoses, with AUC values of 0.639, 0.764, and 0.816 across the three datasets (Fig. 7j–l). Furthermore, this gene set demonstrated predictive capability for the survival of cervical cancer patients in the TCGA database, achieving an AUC of 0.805 (Fig. 7m). These results collectively highlight the utility of the CXCL13^+^ T cell signature as a predictor of tumor recurrence in CESC patients undergoing CCRT and as a marker for immunotherapy efficacy in solid tumors.

## Discussion

Cervical cancer remains a significant global health challenge, with CCRT being the standard treatment for locally advanced stages. Despite initial treatment success, tumor recurrence within 5 years remains a critical concern [9, 17, 18]. In this study, we identified a higher proportion of CXCL13⁺ CD4⁺ and CD8⁺ T cell subsets in pre-treatment tumor tissues, which correlates with recurrence of CSCC.

CXCL13 is a chemokine that plays a crucial role in recruiting B cells and promoting the formation of tertiary lymphoid structures (TLS) within the TME. TLS have been associated with enhanced antitumor immune responses and improved patient outcomes. Previous studies have shown that CXCL13-expressing T cells facilitate B cell recruitment and TLS formation, leading to favorable prognoses in cancers such as breast and lung cancers [16, 19, 20]. Our findings extend these observations to cervical cancer, suggesting that CXCL13⁺ T cells contribute to a robust immune microenvironment that enhances CCRT efficacy and suppresses recurrence. Furthermore, the presence of CXCL13⁺ T cells may indicate a pre-existing immune activation state within the TME, characterized by efficient antigen presentation and immune cell recruitment, thereby priming the tumor for enhanced immune surveillance and therapeutic responses.

Although the prognostic value of CXCL13⁺ T cells is well established, the underlying mechanisms remain to be fully elucidated. CXCL13⁺ T cells are a subset of exhausted T cells characterized by high expression of inhibitory molecules, such as PD-1 and TIM-3, while retaining partial effector functions [21, 22]. Our analysis of cellular communication revealed that signaling pathways such as TGFβ, LCK, and SELPLG were enriched in non-recurrent tumors, consistent with their potential roles in mediating immune responses. Notably, the TGFβ pathway has been shown to upregulate CXCL13 and other immune-associated inhibitory molecules, including PD-1 and TIM-3 [23]. While TGFβ is often considered immunosuppressive, it also plays a critical role in maintaining T cell stemness and function [24, 25]. Similarly, LCK activation is essential for initiating T cell activation and cytokine production, highlighting the functional relevance of these pathways in non-recurrent tumors.

CCRT exerts both cytotoxic effects on tumor cells and immunomodulatory changes in the TME. Radiation therapy, in particular, enhances tumor antigen release, antigen presentation by macrophages and dendritic cells, and T cell infiltration [26, 27]. In this study, we observed that in recurrent tumors, CD163⁺ TAMs interacted with CXCL13⁺ CD8⁺ T cells through the LT and CD39 signaling pathways. TAMs, which are among the most abundant immune cells in the TME, are known to secrete immunosuppressive molecules such as IL-10 and TGFβ, contributing to immune evasion, tissue remodeling, and therapy resistance [28, 29]. The LT pathway, involving LTα and LTβ, is crucial for TLS formation and maintenance, which promotes the generation of CD103⁺ CD8⁺ tissue-resident memory T cells through the LTα-TNFR2 axis [30]. However, the exact mechanisms by which CD163⁺ TAMs influence CXCL13⁺ T cells through the LT pathway remain unclear. The CD39/adenosine signaling pathway represents a key immunosuppressive mechanism in the TME [31]. Our findings revealed that in recurrent tumors, CXCL13⁺ CD8⁺ T cells were the primary emitters of CD39 signals during interactions with myeloid cells [32]. While CD39 expression on T cells has been linked to tumor reactivity and response to immunotherapy [33], hypoxic conditions in the TME can induce high CD39 expression, suppressing T cell effector functions. The dual role of CD39⁺ T cells highlight the complexity of their interactions within the TME. The precise impact of CXCL13⁺ CD8⁺ T cells on CD163⁺ TAMs through this pathway warrants further investigation.

CAFs have been demonstrated to significantly contribute to tumor development, progression, and therapeutic resistance. Previous studies have reported that CAFs contribute to cisplatin resistance in esophageal squamous cell carcinoma by recruiting myeloid-derived suppressor cells [34]. As essential stromal components of tumors, cancer-associated fibroblasts (CAFs) mediate immune cell exclusion, consequently facilitating tumor immune escape [35, 36]. Within the tumor microenvironment, CAFs exhibit heterogeneous phenotypes and perform diverse functions [37]. In this study, we identified robust cell–cell communication between MMP3^+^ CAFs and two CXCL13^+^ T-cell subsets, with the MIF pathway emerging as the predominant signaling mechanism. MIF serves as a crucial molecule in the tumor microenvironment that promotes tumor progression and mediates immune suppression [38]. In esophageal squamous cell carcinoma, inflammatory CAFs interact with tumor cells through the MIF-ACKR3 axis [39]. Our study further suggests that MMP3^+^ CAFs can suppress the functionality of CXCL13^+^ T-cell subsets through MIF secretion.

This study has several limitations. First, the relatively small sample size may require additional evidence to substantiate certain findings. Second, while we analyzed public datasets, the lack of single-cell sequencing data from post-CCRT specimens and the limited sample size constrain our ability to fully elucidate the dynamic changes before and after treatment, which could further explain the differential responses to CCRT.

In conclusion, this study, through single-cell RNA sequencing, reveals that the pre-existing proportion of CXCL13⁺ T cell subsets in tumor tissues is positively associated with non-recurrence following CCRT. Furthermore, our analysis of T cell and myeloid cell interactions uncovered potential mechanisms through which CXCL13⁺ T cells contribute to antitumor immunity. Finally, we developed a five-gene random forest model based on CXCL13⁺ T cell gene signatures, which effectively predicts recurrence in CESC patients treated with CCRT and response to immunotherapy in solid tumors. These findings underscore the prognostic and therapeutic potential of CXCL13⁺ T cells in cervical cancer and beyond.

## Supplementary Information

Below is the link to the electronic supplementary material.

Supplementary file 1. Supplementary Fig. 1: scRNA-seq data filtering and integration. a, b Violin plot showing the expression of feature and count of RNA, and proportions of RP (pRP), MT (pMT) and HB (pHB) genes in each sample before filtering. b Scatter plot showing correlation of nFeature_RNA, nCount_RNA，(pRP), (pMT) and (pHB). c Violin plot showing the expression of feature and count of RNA, and pRP, pMT and pHB genes in each sample after filtering. d PCA analysis of each sample before (upper) and after (lower) integration. e Violin plot showing the expression of cell cycle (S phase and G2M phase) score and the difference between S score and G2M score. (TIF 28467 kb)

Supplementary file 2. Supplementary Fig. 2: Characterization of major cell subsets. a UMAP plots showing clusters derived from six CESC tumor tissues. b Violin plot showing the expression of canonical marker genes of major cell types. c UMAP plots showing cell types derived from six CESC tumor tissues prior and post CCRT analyzed using GSE236738 scRNA-seq data. d Violin plot showing the expression of canonical marker genes of major cell types in GSE236738 scRNA-seq data. e Dot plot showing the top 5 maker genes of each cell type in GSE236738 scRNA-seq data. (TIF 16960 kb)

Supplementary file 3. Supplementary Fig. 3: Distribution of major cell types. a UMAP plot showing the 12 cell subsets separated by samples. b Boxplot showing the ratios of major cell subsets between recurrent and non-recurrent tumors. (TIF 17642 kb)

Supplementary file 4. Supplementary Fig. 4: Characterization of T cell subsets. a UMAP plots showing expression of CD3D, CD4 and CD8A. b UMAP plot showing T cell subsets separated by samples. c Dot plot showing functional gene expression, including cytotoxic, co-stimulatory，chemokine and receptor and co-inhibitory genes, in CD4+ T cells. d Boxplots showing the distribution of 15 cell subsets of T cells in recurrent and non-recurrent tumors, respectively. e Boxplot showing the ratios of T cell subsets between recurrent and non-recurrent tumors. (TIF 32995 kb)

Supplementary file 5. Supplementary Fig. 5. Comparison of CXCL13+ CD4+ and CD8+ T cell subsets between pre- and post-CCRT treatment groups. a UMAP plots showing subsets of CD8+ T cells and CD4+ T cells using GSE236738 scRNA-seq data. b Dot plot showing the top 5 maker genes of each subset of CD8+ T cells and CD4+ T cells. c Boxplot showing the comparison of CD8-C7-CXCL13 and CD4-C6-CXCL13 subset prior and after CCRT. (TIF 19004 kb)

Supplementary file 6. Supplementary Fig. 6: Characterization of myeloid cell subsets. a UMAP plots showing expression of LYZ, C1QA, C1QB, CD1C, S100A8 and S100A9. b UMAP plot showing T cell subsets separated by samples. c UMAP plot showing T cell subsets separated by recurrent status. d Boxplot showing the ratios of myeloid cell subsets between recurrent and non-recurrent tumors. (TIF 20606 kb)

Supplementary file 7. Supplementary Fig. 7: Interactions within myeloid cell subsets. a Bar plot showing the number and strength of interactions across myeloid cell subsets in recurrent and non-recurrent tumors. b Stacked plot showing different signaling in recurrent and non-recurrent tumors medicated interactions across myeloid cell subsets. (TIF 11359 kb)

Supplementary file 8. Supplementary Fig. 8: Interactions of CXCL13+ T cells and myeloid subsets. a Intersections of specific incoming and outgoing signaling between c3-CXCL13-CD4(Tfh) and c10-CXCL13-CD8(Tex) in recurrent and non-recurrent tumors, respectively. b Hierarchy plot showing receivers and senders of OX40 signaling specific to c10-CXCL13-CD8(Tex) subset in recurrent tumors. c, d Hierarchy plot showing receivers and senders of IL-2, SELPLG and IL-16 signaling specific to c3-CXCL13-CD4(Tfh) subset in recurrent tumors. e Hierarchy plot showing receivers and senders of ALCAM, CD6, CXCL, OSM and SN signaling specific to c3-CXCL13-CD4(Tfh) subset in non-recurrent tumors. (DOCX 14 kb)

## Data Availability

The raw single-cell RNA sequencing data are available in the GEO database under accession number GSE224327. Bulk RNA-seq data were obtained from the online website UCSCxena (http://xena.ucsc.edu/). Other data used in the study are available from the corresponding author upon reasonable request.
