# Supplementary figures and images for: Single-cell RNA sequencing analysis reveals a lack of CXCL13+ T cell subsets associated with the recurrence of cervical squamous cell carcinoma following concurrent chemoradiotherapy
Source: Cancer Immunol Immunother. 2025 Jun 4;74(7):235. doi: 10.1007/s00262-025-04083-3 (PMC12137842; doi:10.1007/s00262-025-04083-3)

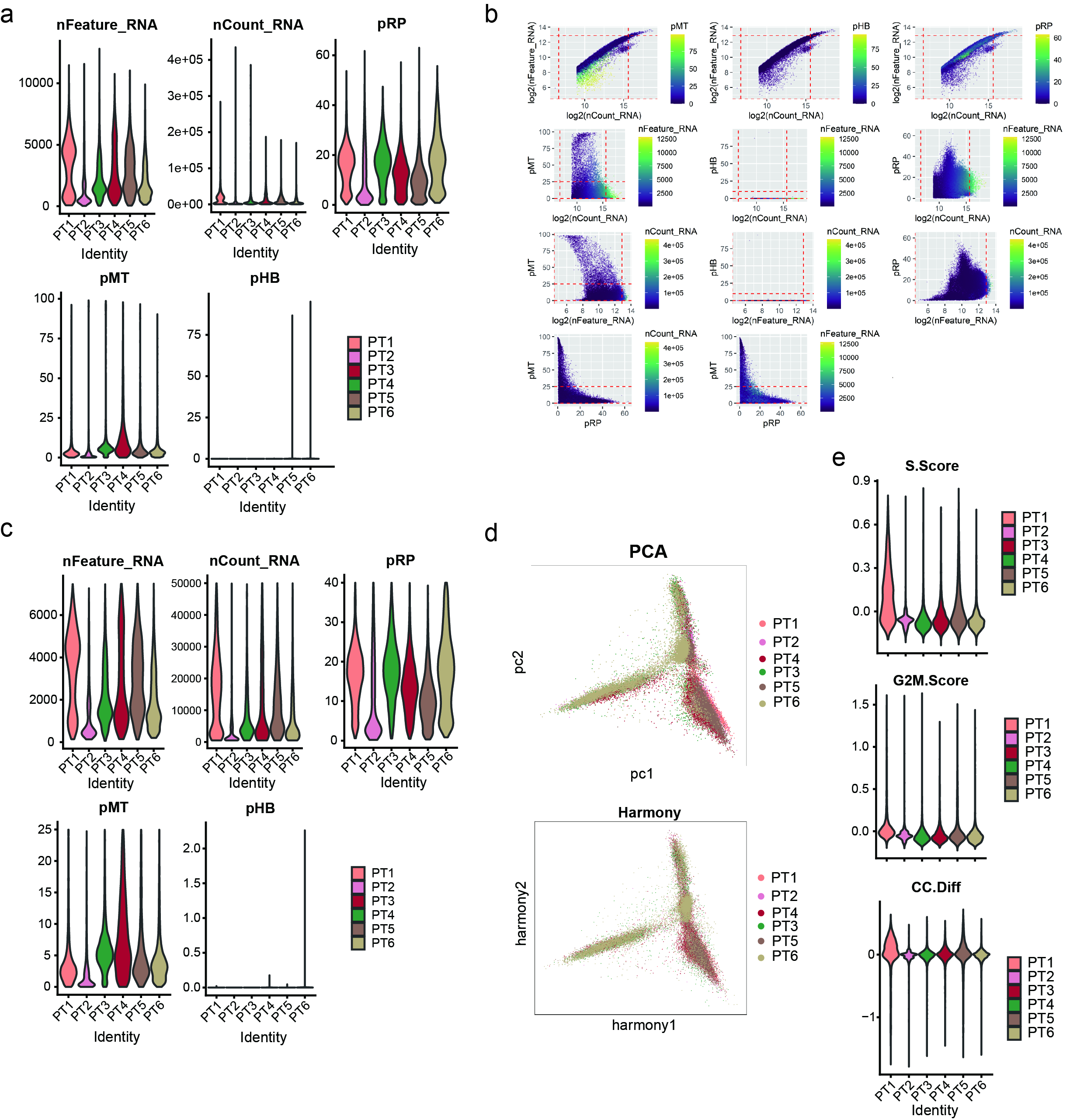

Supplement: Supplementary file 1 — Supplementary Fig. 1: scRNA-seq data filtering and integration. a, b Violin plot showing the expression of feature and count of RNA, and proportions of RP (pRP), MT (pMT) and HB (pHB) genes in each sample before filtering. b Scatter plot showing correlation of nFeature_RNA, nCount_RNA，(pRP), (pMT) and (pHB). c Violin plot showing the expression of feature and count of RNA, and pRP, pMT and pHB genes in each sample after filtering. d PCA analysis of each sample before (upper) and after (lower) integration. e Violin plot showing the expression of cell cycle (S phase and G2M phase) score and the difference between S score and G2M score. (TIF 28467 kb) [file 262_2025_4083_MOESM1_ESM.tif]

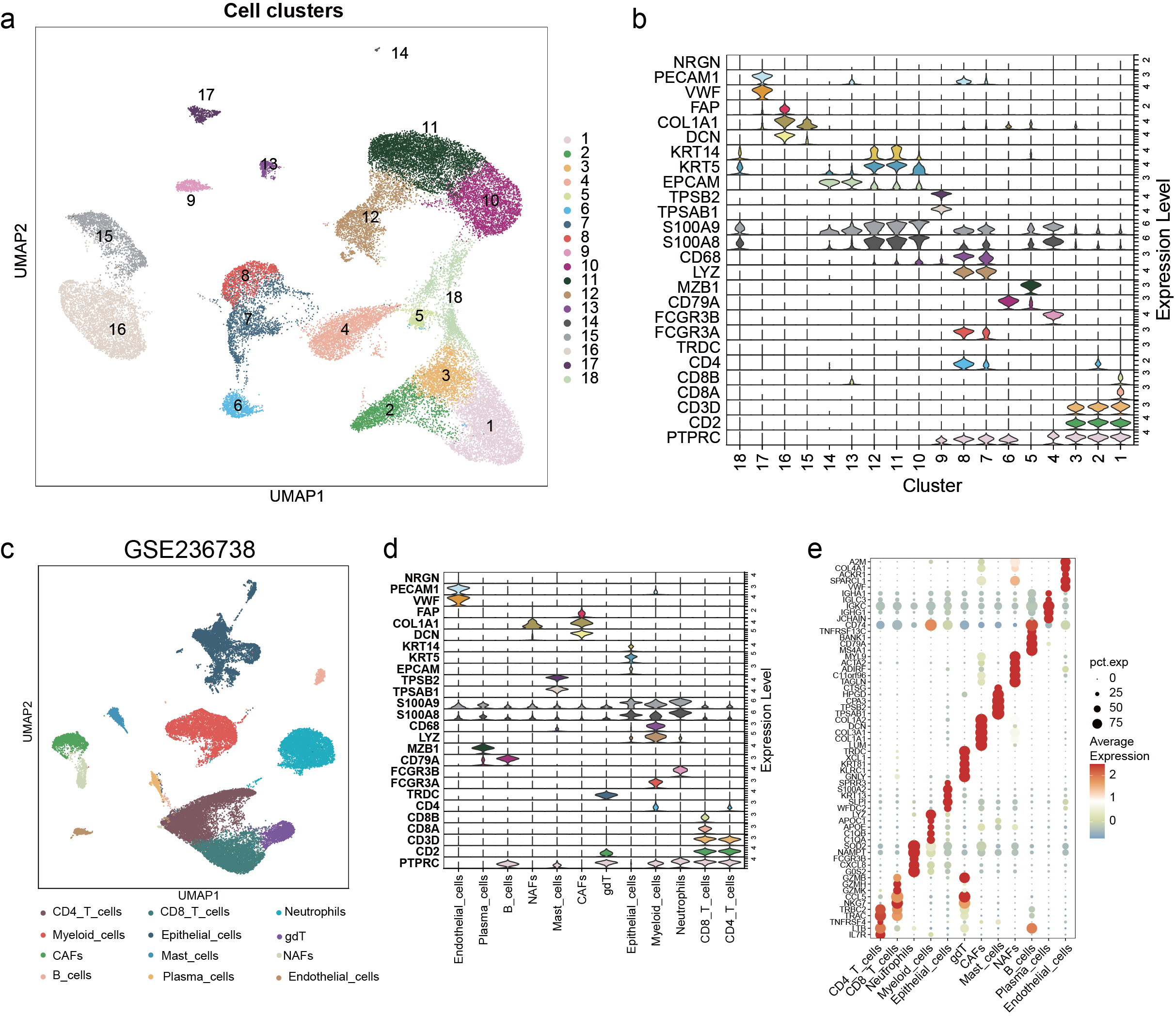

Supplement: Supplementary file 2 — Supplementary Fig. 2: Characterization of major cell subsets. a UMAP plots showing clusters derived from six CESC tumor tissues. b Violin plot showing the expression of canonical marker genes of major cell types. c UMAP plots showing cell types derived from six CESC tumor tissues prior and post CCRT analyzed using GSE236738 scRNA-seq data. d Violin plot showing the expression of canonical marker genes of major cell types in GSE236738 scRNA-seq data. e Dot plot showing the top 5 maker genes of each cell type in GSE236738 scRNA-seq data. (TIF 16960 kb) [file 262_2025_4083_MOESM2_ESM.tif]

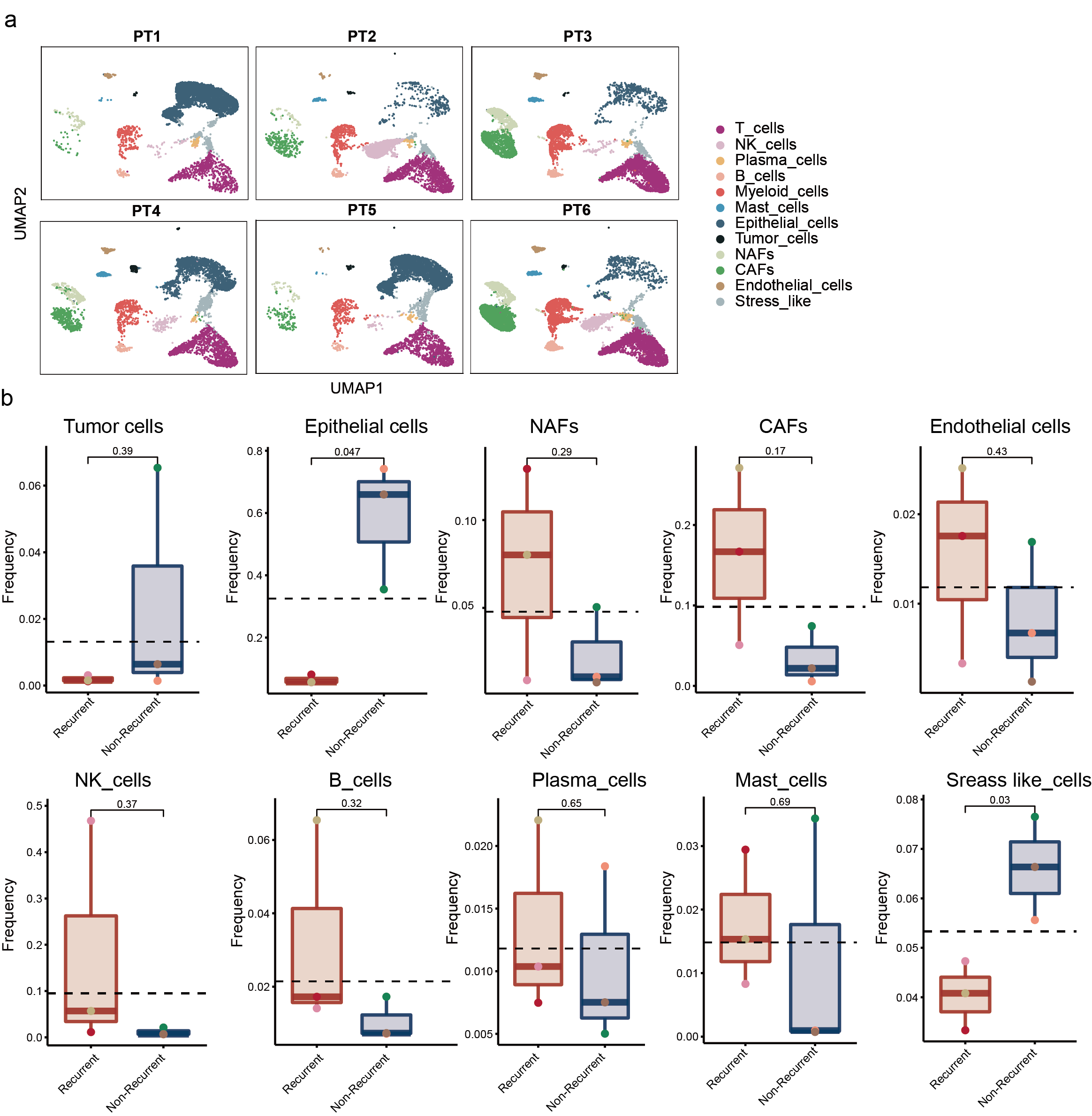

Supplement: Supplementary file 3 — Supplementary Fig. 3: Distribution of major cell types. a UMAP plot showing the 12 cell subsets separated by samples. b Boxplot showing the ratios of major cell subsets between recurrent and non-recurrent tumors. (TIF 17642 kb) [file 262_2025_4083_MOESM3_ESM.tif]

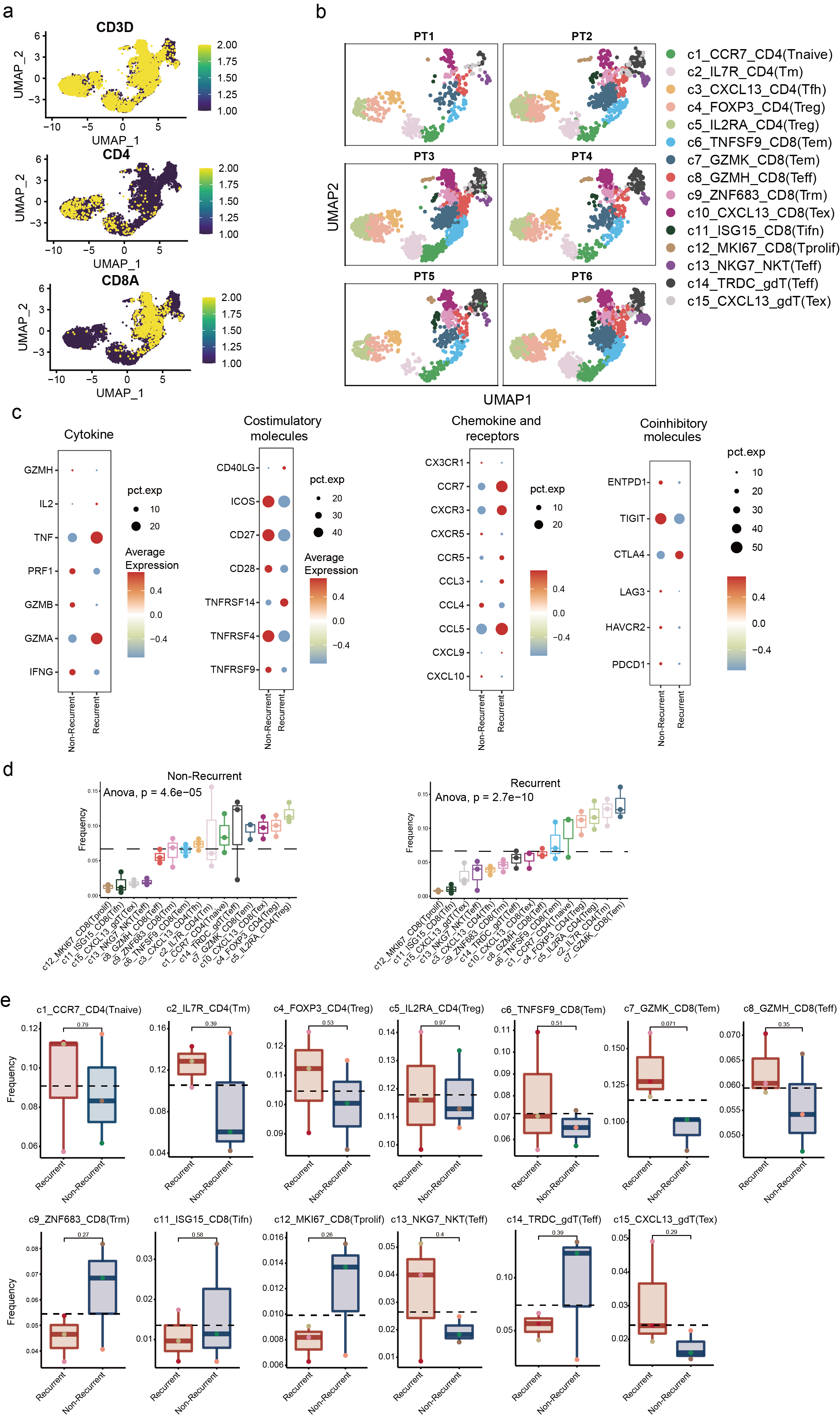

Supplement: Supplementary file 4 — Supplementary Fig. 4: Characterization of T cell subsets. a UMAP plots showing expression of CD3D, CD4 and CD8A. b UMAP plot showing T cell subsets separated by samples. c Dot plot showing functional gene expression, including cytotoxic, co-stimulatory，chemokine and receptor and co-inhibitory genes, in CD4+ T cells. d Boxplots showing the distribution of 15 cell subsets of T cells in recurrent and non-recurrent tumors, respectively. e Boxplot showing the ratios of T cell subsets between recurrent and non-recurrent tumors. (TIF 32995 kb) [file 262_2025_4083_MOESM4_ESM.tif]

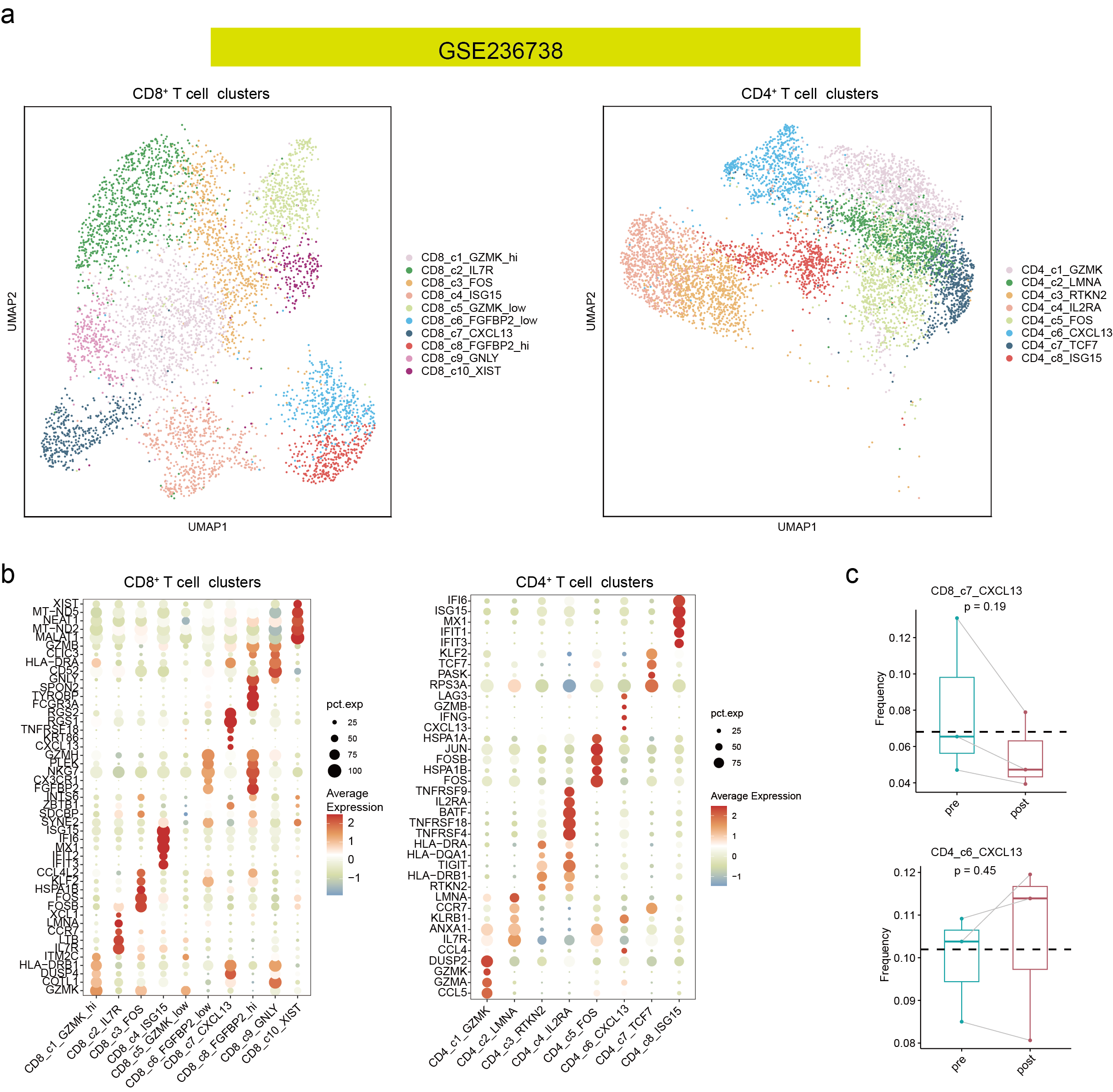

Supplement: Supplementary file 5 — Supplementary Fig. 5. Comparison of CXCL13+ CD4+ and CD8+ T cell subsets between pre- and post-CCRT treatment groups. a UMAP plots showing subsets of CD8+ T cells and CD4+ T cells using GSE236738 scRNA-seq data. b Dot plot showing the top 5 maker genes of each subset of CD8+ T cells and CD4+ T cells. c Boxplot showing the comparison of CD8-C7-CXCL13 and CD4-C6-CXCL13 subset prior and after CCRT. (TIF 19004 kb) [file 262_2025_4083_MOESM5_ESM.tif]

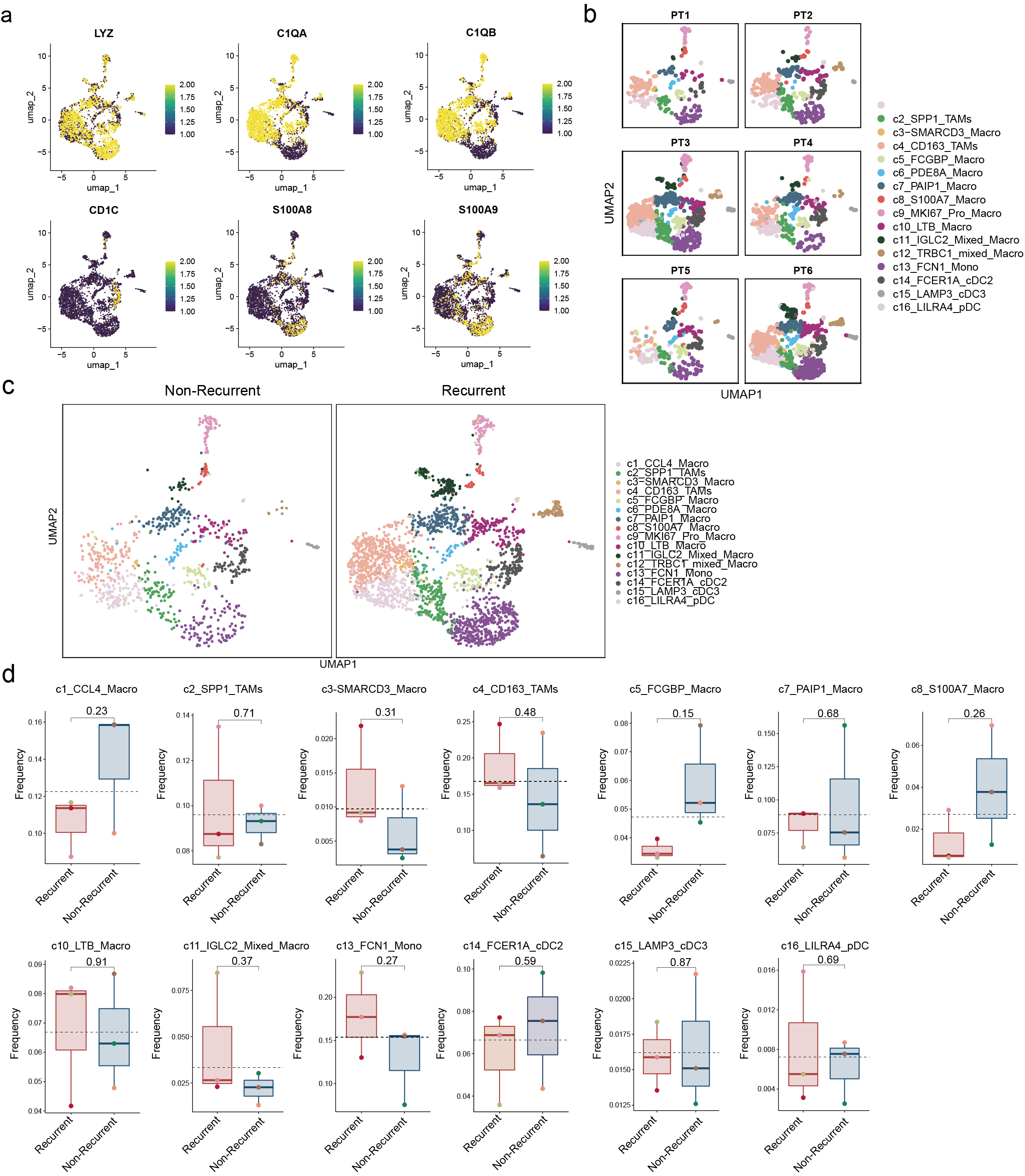

Supplement: Supplementary file 6 — Supplementary Fig. 6: Characterization of myeloid cell subsets. a UMAP plots showing expression of LYZ, C1QA, C1QB, CD1C, S100A8 and S100A9. b UMAP plot showing T cell subsets separated by samples. c UMAP plot showing T cell subsets separated by recurrent status. d Boxplot showing the ratios of myeloid cell subsets between recurrent and non-recurrent tumors. (TIF 20606 kb) [file 262_2025_4083_MOESM6_ESM.tif]

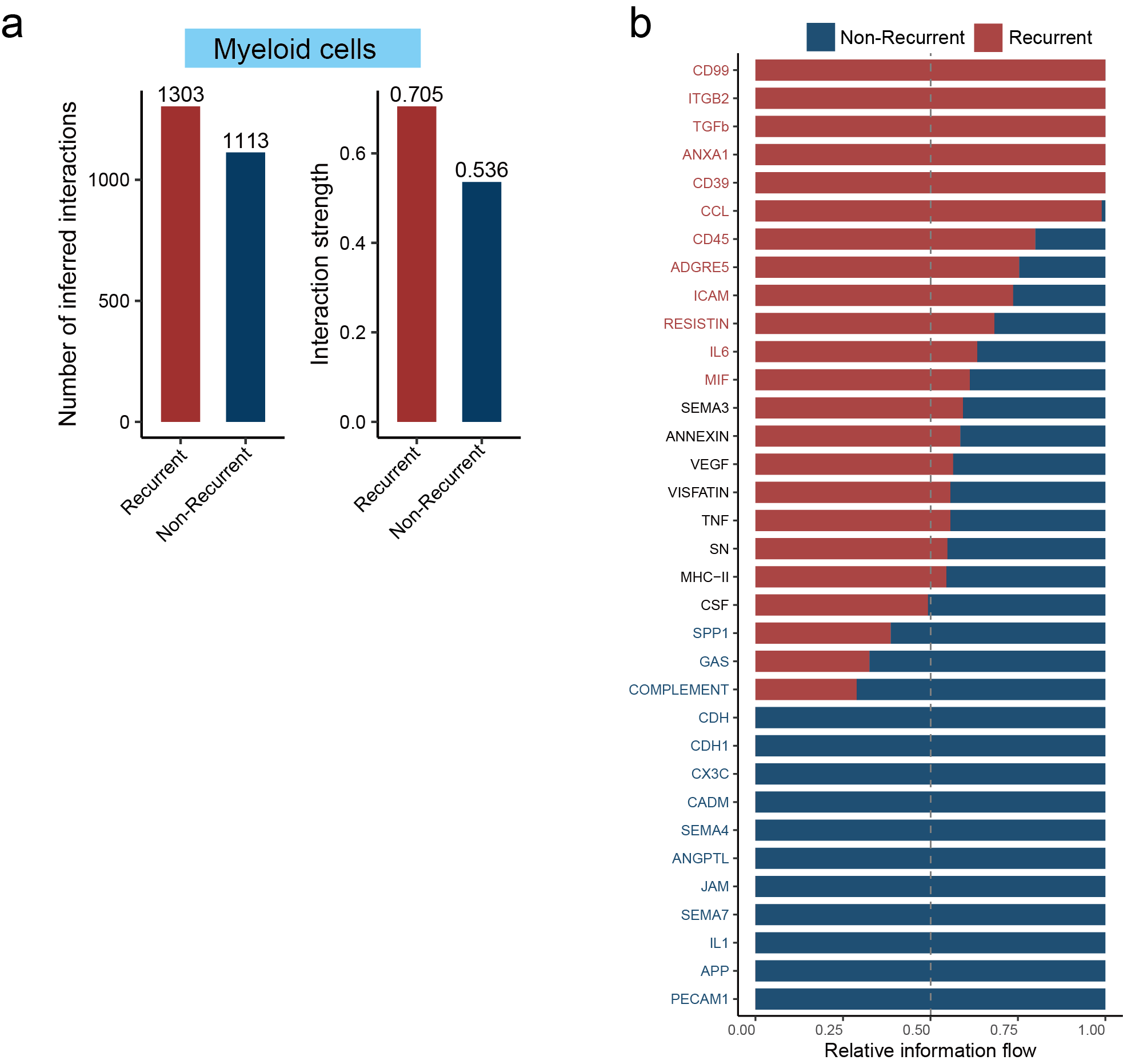

Supplement: Supplementary file 7 — Supplementary Fig. 7: Interactions within myeloid cell subsets. a Bar plot showing the number and strength of interactions across myeloid cell subsets in recurrent and non-recurrent tumors. b Stacked plot showing different signaling in recurrent and non-recurrent tumors medicated interactions across myeloid cell subsets. (TIF 11359 kb) [file 262_2025_4083_MOESM7_ESM.tif]
