# Supplementary material for: Single-cell RNA sequencing analysis reveals a lack of CXCL13+ T cell subsets associated with the recurrence of cervical squamous cell carcinoma following concurrent chemoradiotherapy
Source: Cancer Immunol Immunother. 2025 Jun 4;74(7):235. doi: 10.1007/s00262-025-04083-3 (PMC12137842; doi:10.1007/s00262-025-04083-3)
Supplement: Supplementary file 8 — Supplementary Fig. 8: Interactions of CXCL13+ T cells and myeloid subsets. a Intersections of specific incoming and outgoing signaling between c3-CXCL13-CD4(Tfh) and c10-CXCL13-CD8(Tex) in recurrent and non-recurrent tumors, respectively. b Hierarchy plot showing receivers and senders of OX40 signaling specific to c10-CXCL13-CD8(Tex) subset in recurrent tumors. c, d Hierarchy plot showing receivers and senders of IL-2, SELPLG and IL-16 signaling specific to c3-CXCL13-CD4(Tfh) subset in recurrent tumors. e Hierarchy plot showing receivers and senders of ALCAM, CD6, CXCL, OSM and SN signaling specific to c3-CXCL13-CD4(Tfh) subset in non-recurrent tumors. (DOCX 14 kb) [file 262_2025_4083_MOESM8_ESM.docx]

| Gene | Forward primers | Reverse primers |
| --- | --- | --- |
| PDCD1 | CCAGGATGGTTCTTAGACTCCC | TTTAGCACGAAGCTCTCCGAT |
| CXCL13 | GCTTGAGGTGTAGATGTGTCC | CCCACGGGGCAAGATTTGAA |
| TOX | TATGAGCATGACAGAGCCGAG | GGAAGGAGGAGTAATTGGTGGA |
| RGS1 | TCTTCTCTGCTAACCCAAAGGA | TGCTTTACAGGGCAAAAGATCAG |
| ALOX5AP | TCAGCGTGGTCCAGAATGG | GCAAGTGTTCCGGTCCTCT |

**Supplementary Table 1:** Primer sequences of genes used in this study.
